# First in-human radiation dosimetry of ^68^Ga-NODAGA-RGDyK

**DOI:** 10.1186/s13550-017-0288-x

**Published:** 2017-05-18

**Authors:** Silvano Gnesin, Periklis Mitsakis, Francesco Cicone, Emmanuel Deshayes, Vincent Dunet, Augusto F. Gallino, Marek Kosinski, Sébastien Baechler, Franz Buchegger, David Viertl, John O. Prior

**Affiliations:** 1 Institute of Radiation Physics, Lausanne University Hospital, Rue du Bugnon 45, CH-1011 Lausanne, Switzerland; 2 Department of Nuclear Medicine and Molecular Imaging, Lausanne University Hospital, Lausanne, Switzerland; 3 Nuclear Medicine, Department of Surgical and Medical Sciences and Translational Medicine, “Sapienza” University of Rome, Rome, Italy; 4 Institut régional du Cancer de Montpellier, Montpellier, France; 5 Department of Radiodiagnostic and Interventional Radiology, Lausanne University Hospital, Lausanne, Switzerland; 6 Department of Cardiology, Ospedale San Giovanni, Bellinzona, Switzerland

**Keywords:** Angiogenesis, Dosimetry, PET/CT, ^68^Ga-NODAGA-RGDyK, Choroid plexuses, Integrin α_v_β_3_

## Abstract

**Background:**

Integrin-targeting radiopharmaceuticals have potential broad applications, spanning from cancer theranostics to cardiovascular diseases. We have previously reported preclinical dosimetry results of ^68^Ga-NODAGA-RGDyK in mice. This study presents the first human dosimetry of ^68^Ga-NODAGA-RGDyK in the five consecutive patients included in a clinical imaging protocol of carotid atherosclerotic plaques. Five male patients underwent whole-body time-of-flight (TOF) PET/CT scans 10, 60 and 120 min after tracer injection (200 MBq). Quantification of ^68^Ga activity concentration was first validated by a phantom study. To be used as input in OLINDA/EXM, time-activity curves were derived from manually drawn regions of interest over the following organs: brain, thyroid, lungs, heart, liver, spleen, stomach, kidneys, red marrow, pancreas, small intestine, colon, urinary bladder and whole body. A separate dosimetric analysis was performed for the choroid plexuses. Female dosimetry was extrapolated from male data. Effective doses (EDs) were estimated according to both ICRP60 and ICRP103 assuming 30-min and 1-h voiding cycles.

**Results:**

The body regions receiving the highest dose were urinary bladder, kidneys and choroid plexuses. For a 30-min voiding cycle, the EDs were 15.7 and 16.5 μSv/MBq according to ICRP60 and ICRP103, respectively. The extrapolation to female dosimetry resulted in organ absorbed doses 17% higher than those of male patients, on average.

The 1-h voiding cycle extrapolation resulted in EDs of 19.3 and 19.8 μSv/MBq according to ICRP60 and ICRP103, respectively. A comparison is made with previous mouse dosimetry and with other human studies employing different RGD-based radiopharmaceuticals.

**Conclusions:**

According to ICRP60/ICRP103 recommendations, an injection of 200 MBq ^68^Ga-NODAGA-RGDyK leads to an ED in man of 3.86/3.92 mSv. For future therapeutic applications, specific attention should be directed to delivered dose to kidneys and potentially also to the choroid plexuses.

**Trial registration:**

Clinical trial.gov, NCT01608516

## Background

The development of new blood vessels, namely neo-angiogenesis, plays an essential role in many physiological and pathological processes. In oncology, factors mediating endothelial cell adhesion and migration (e.g. integrins), as well as vascular growth factors, are associated with tumour progression and development of metastases, and have become the target of several anti-cancer compounds [1].

A specific marker of angiogenesis is the a_v_β_3_ integrin, which is targeted with high affinity by the peptidic compound arginine-glycine-aspartic acid (RGD). Several proteins of the extracellular matrix like vitronectin, fibrinogen and fibronectin interact with integrin α_ν_β_3_ via the RGD sequence [2].

A number of single photon (SPECT) and positron emitting (PET) tracers for α_ν_β_3_ integrin have been developed based on RGD cyclic sequences, such as RGDyK or RGDfK [3]. Among the PET tracers, ^18^F-Galacto-RGD has been most extensively used but its synthesis has a low radiochemical yield and requires almost two ^18^F half-lives, on average [4]. Radiolabelling of generator-produced ^68^Ga with either DOTA or NODAGA chelators allows for a more efficient, faster and fully automated production [5–9].

Many of these integrin-targeting radiolabelled compounds, initially developed for cancer theranostics, have been later applied to cardiovascular diseases, showing that neo-angiogenesis is a quantifiable process of cardiac remodeling after an ischemic injury in animal models [10–12] as well as in humans [13, 14].

Since integrin-mediated cell chemotaxis and angiogenesis represent a key feature of atherosclerotic plaque vulnerability [15], radionuclide imaging of α_ν_β_3_ can be applied earlier in the ischemic process, potentially identifying those vascular lesions carrying an increased risk of complications [16–20].

Carotid artery disease is one of the leading causes of morbidity and mortality worldwide and represents therefore a field of potential broad application for RGD-based radionuclide probes [19, 20]. Indeed, newer risk models for carotid plaque evaluation include the assessment of vulnerability beyond the degree of luminal narrowing [21–23].

We have previously reported preclinical dosimetry and toxicity results of ^68^Ga-NODAGA-RGDyK in outbred female Hsd ICR (CD-1®) mice [24].

The aim of the present study was to assess the human dosimetry of ^68^Ga-NODAGA-RGDyK in the first five patients included in a clinical research protocol applying multimodality imaging to carotid atherosclerotic plaques before endarterectomy.

## Methods

### Patients

The present dosimetry sub-study was designed to include the first five consecutive patients enrolled in a clinical study comparing ^68^Ga-NODAGA-RGDyK PET/CT, ^18^F-fluorodeoxyglucose (FDG) PET/CT, magnetic resonance and ultrasound against histology in the assessment of carotid plaque vulnerability. Patients were included if presenting with any of the following: (i) symptomatic carotid stenosis of any degree or (ii) asymptomatic stenosis >70%.

Contraindications to surgery or gadolinium injection represented exclusion criteria, together with pregnancy, age >85, Karnofsky index <80% and inability to provide written informed consent.

The study was authorized by the Ethical Committee of Canton Vaud, Swissmedic and the Federal Office of Public Health (FOPH). Patients gave separate written informed consent to the clinical and the dosimetry protocol before radiopharmaceutical administration.

### Radiochemistry

The NODAGA-RGDyK is a GMP-produced monomeric integrin-targeting peptide purchased from Advanced Biochemical compounds ABX, Radeberg, Germany. As previously described [24], the NODAGA-RGDyK was radiolabelled with the ^68^Ga eluate of a ^68^Ge-generator IGC100 (Eckert & Ziegler, Germany) using an automatic processor unit, Modular-Lab PharmTracer (Eckert & Ziegler, Germany). ^68^Ga was eluted with 0.1 mol/l HCl. NODAGA-RGDyK (20 μg) was radiolabelled with the high activity ^68^Ga fraction by incubation for 20 min at room temperature. After cartridge purification, the ready for use ^68^Ga-NODAGA-RGDyK was eluted in 50% ethanol through a 0.22-μm sterile filter and diluted into NaCl solution. High-pressure liquid chromatography analysis was performed on a μ-Bondapak column (Waters C-18) run with trifluoroacetic acid and acetonitrile.

### PET/CT acquisition protocol

Three whole-body images (from top of the skull to mid femora, 2 min/bed position) were acquired on a Discovery 690 time of flight (TOF) PET/CT (GE Healthcare, Milwaukee, Wisconsin, USA) [25] 10 ± 2, 60 ± 5 and 120 ± 5 min after the intravenous injection of 200 MBq ^68^Ga-NODAGA-RGDyK. Patients were asked to void between scans (30-min voiding cycle). The list-mode acquisition integrating TOF information and point-spread-function recovery was reconstructed with a proprietary three-dimension ordered subset expectation maximization (3D-OSEM) algorithm (GE-VPFXS, 3 iterations × 16 subsets) including a FWHM = 5 mm Gaussian post-reconstruction filter. All pertinent image corrections (normalization, dead time, activity decay, random coincidence, attenuation and scatter corrections) were applied. The acquired field of view size was 70 cm reconstructed in a 256 × 256 image matrix. Reconstructed voxel size was 2.73 × 2.73 mm in the transverse plane and 3.27 mm in the axial direction. Morphologic information was obtained from a whole-body CT scan: 120 kVp, 60 mA, pitch = 3. The activity concentration measured in each patient multi-bed acquisition was decay corrected (physical decay correction) to the start of the PET acquisition.

### Quantitative accuracy assessment

Quantitative accuracy was assessed in a NEMA NU2 phantom with the same acquisition and reconstruction parameters used for patients. The phantom (volume 9340 cm^3^) was filled with 42.3 MBq of ^68^Ga in aqueous solution measured at the image acquisition start time, resulting in an activity concentration of 4.52 kBq/mL which mimics the typical average activity concentration present in patient organs. In 10 phantom background regions of 3 × 3 cm^2^, an average activity concentration of 4.25 kBq/mL was measured, which is 6% lower than the actual value. Such discrepancy is compatible with the accuracy of the well counter used for the ^68^Ga activity measurement before filling the phantom.

### Organ segmentation and dose estimations

Volumes of interest (VOI) were manually drawn slice by slice on the axial plane of the CT part of every PET/CT study using PMOD (PMOD Technologies, Zurich, Switzerland) for the following body regions: brain, thyroid, lungs, heart, liver, spleen, stomach, kidneys, red marrow, pancreas, small intestine, colon and whole body. Choroid plexuses and urinary bladder were visually segmented on the emission PET data by two operators in consensus (SG and FC). CT-based segmentation of choroid plexuses would have been challenging and, if applied to bladder, it would have not taken into account possible changes of volume due to bladder filling during the PET/CT acquisition time.

As recommended by the MIRD pamphlet 16 for a three-data point study [26], a mono-exponential fit extended to infinite beyond the last measured data point was used to derive time-integrated activity coefficients (TIAC) in each selected organ. The goodness of fit for each organ was expressed by the *R* ^2^ metric. Organ residence times were obtained by dividing TIAC for the administered ^68^Ga total activity. For each patient, two red marrow VOIs were drawn, the first in the heads of both femora and the second in the lumbar vertebrae L3–L4. The total number of disintegration which occurred in the red marrow was obtained by averaging the number of disintegration per unit of mass of these two regions and then multiplying this value by the red marrow mass of the OLINDA adult male reference phantom [27]. This methodology is an extension to 3D images of the method presented by Ferrer et al. [28]. The calculation performed in two separate bone marrow regions, the lumbar vertebrae (L3–L4) and the head of femora aimed at reducing quantitative bias due to heterogeneity in red marrow uptake [29]. Since no relevant heterogeneities of activity distribution were observed in the colon tract, the total number of disintegrations in this organ was partitioned to its components (upper large intestine, lower large intestine) proportionally to their respective masses of the OLINDA male reference phantom. Organ residence times were used in input to the OLINDA/EXM (Organ Level INternal Dose Assessment/EXponential Modeling) code [30]. OLINDA/EXM provided organ absorbed doses and effective dose (ED) per absorbed activity in μGy/MBq and μSv/MBq, respectively, using the ICRP60 tissue weighting factors [31]. The ED was also re-evaluated manually from organ equivalent doses adopting the tissue weighting factors recommended by the ICRP103 [32]. The influence of a longer urinary bladder voiding cycle (1 h) on dose estimations was also assessed to facilitate the comparison with previous similar dosimetric studies of RGD-like radiopharmaceuticals [33–37]

A specific dose assessment was performed for the choroid plexuses, which do not appear in the list of available organs in OLINDA. The residence time measured for this VOI was used as input in the OLINDA sphere model. The mass of the body, atrial and temporal part of the choroid plexuses in the lateral ventricles was estimated assuming a cylindrical geometry based on their length and width reported by Kuruoglu et al. for both male and female subjects [38]. The masses of the choroid plexuses were estimated to be 2.76 and 1.81 g for male and female, respectively, which is close to the mass of 2 g reported by others [39].

Having enrolled only male patients, the organ dose and ED for female were extrapolated from male reference times applied to the organ masses of the OLINDA/EXM adult female phantom, considering a voiding cycle of 1 h. The ED for the reference person was computed from the equivalent organ dose for male and female according to the ICRP 103 methodology.

## Results

### Imaging

Five male patients (mean age 66, range 53–76 years), were included. Three patients presented with symptoms associated with carotid stenosis, the two remaining patients were asymptomatic. Patient characteristics, including severity and site of carotid artery stenosis, are reported in Table 1.

**Table 1:** Patient characteristics, including neurological symptoms and % stenosis, as measured by magnetic resonance imaging (MRI)

Patient ID	Age (years)	Body mass index (kg/m^2^)	Symptoms (Y/N)	Diagnosis (% stenosis)
1	62	30	N	>80% right ICA^a^
2	53	23	Y	84% right ICA; 65% left ICA
3	65	26	Y	80% right ICA^a^
4	76	31	N	>90% left ICA; >50% right ICA
5	72	21	Y	>80% left ICA^a^

*ICA* internal carotid artery

^a^Patients who underwent previous contralateral endarterectomy

At the time of writing, definitive results of the main clinical protocol are not available and will be reported elsewhere. A preliminary comparison between ^68^Ga-NODAGA-RGDyK and FDG PET/CT suggests a higher rate of lesion detection for ^68^Ga-NODAGA-RGDyK, with 3/5 sites of carotid stenosis showing increased uptake values compared with the controlateral side. In contrast, FDG was unremarkable at the sites of known stenosis. An increased FDG uptake was seen in the soft tissues at the site of previous surgery in all the three patients who had undergone contralateral endarterectomy 2 to 12 months before the start of the current imaging protocol.

### Patient dosimetry

**Fig. 1:**
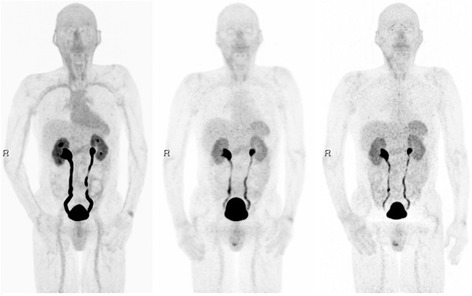
From l*eft* to *right*, example of maximum intensity projections 10, 60 and 120 min post tracer injection showing ^68^Ga-NODAGA-RGDyK uptake distribution in major abdominal organs. In this patient case, retention of radio-urine in the excretory system is exacerbated by prostatic hypertrophy

**Fig. 2:**
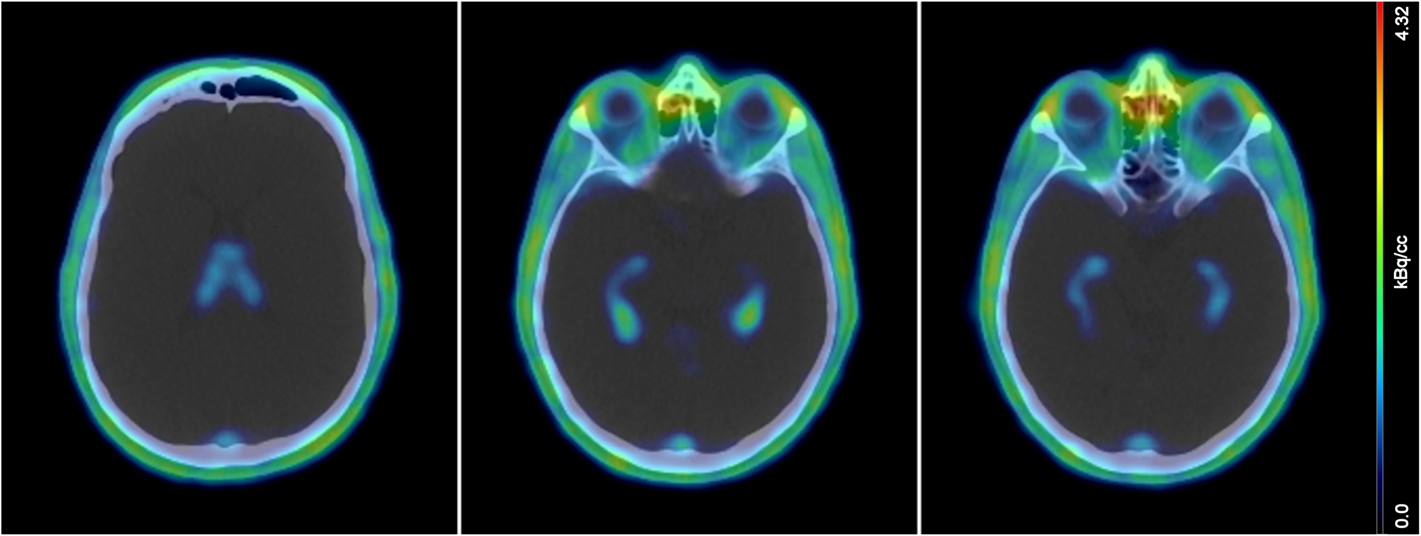
Typical uptake pattern in the choroid plexuses of the lateral ventricles. *Left-hand site* panel shows ^68^Ga-NODAGA-RGDyK absence of uptake in the frontal horns of the lateral ventricles. The *central* and *right-hand side* panels show increased uptake in the occipital and temporal horns of the lateral ventricles, while only faint uptake is seen in the plexus of the third ventricle

**Fig. 3:**
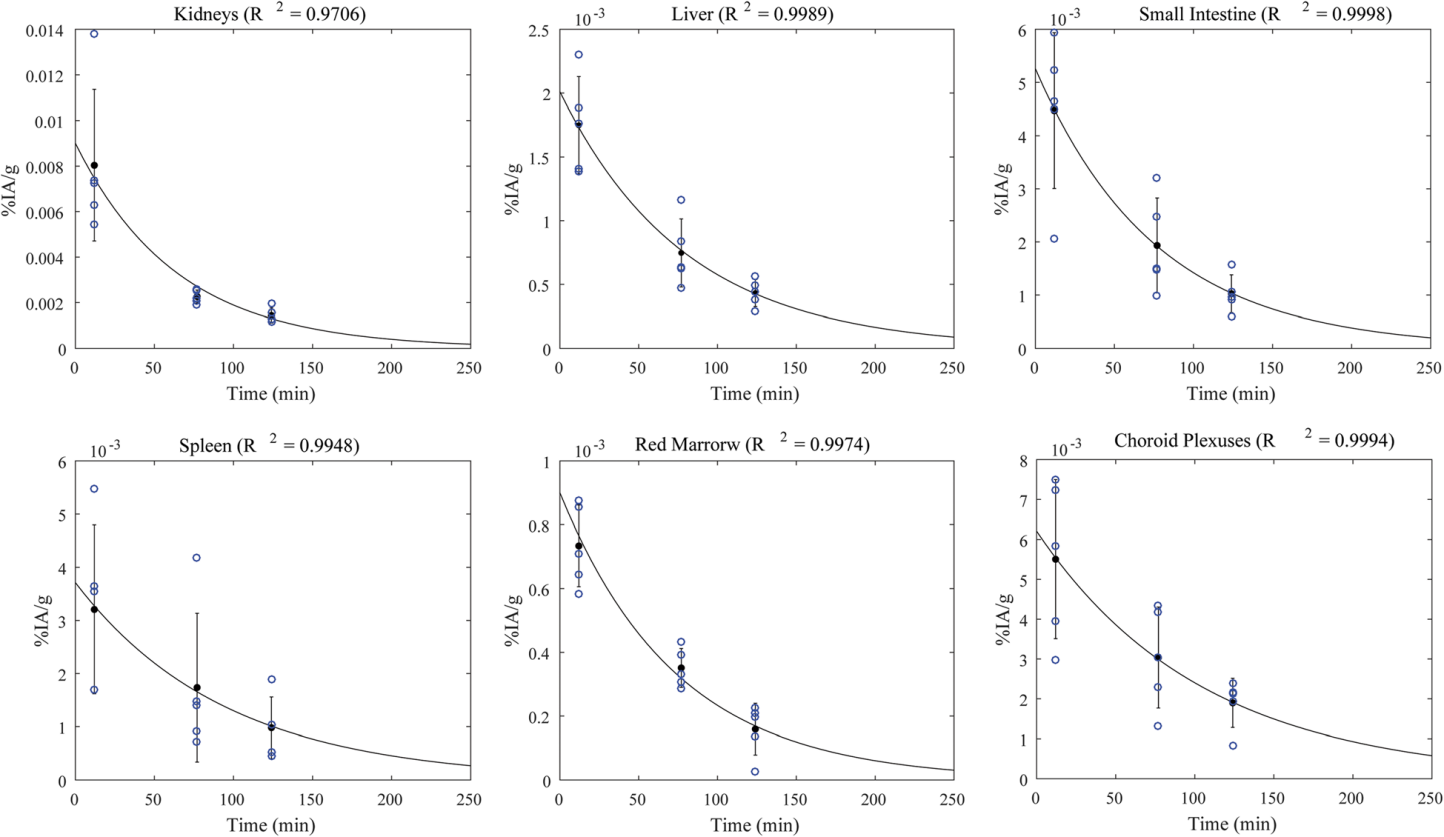
^68^Ga-NODAGA-RGDyK time-activity concentrations for some of the most irradiated organs. The organ activity was normalized to the injected activity and organ mass (%IA/g). *Blue circles* at each time point indicates the measurements for the five patients enrolled in this study. *Solid lines* represent the mono-exponential fits and *error bars* indicate ±SD. The coefficient of determination (*R* ^2^) of the fit is reported for each organ

All injections were well-tolerated. No immediate symptoms or modification of vital signs were observed. At first time point images, significant uptake was seen in the urinary system. Mild uptake was also observed in the thyroid gland, liver, spleen and intestine. The kidney activity showed only a mild decrease over time, being still visible at the latest time point images. Figure 1 shows an example of maximum intensity projection images obtained 10, 60 and 120 min after tracer injection. A significant uptake was observed in the choroid plexuses since the early time point images, as already described by others [35] (see Fig. 2). Time-activity curves for kidneys, liver, small intestine, spleen, red marrow and choroidal plexuses are shown in Fig. 3.

The residence times, organ absorbed doses and ED of the patients enrolled, as well as the extrapolated values to female and reference person, are reported in Table 2.

**Table 2:** Residence times, organ absorbed doses and EDs according ICRP60 and ICRP103 for 30-min and 1-h urinary voiding cycles in men. Extrapolated organ dose in female and defence person is reported for 1-h urinary voiding cycle only

			Patient cohort (*n* = 5 male)				Female	Reference
	Residence time		0.5-h voiding		1-h voiding		1-h voiding	
Target organ	Mean (hours)	SD	Dose (mGy/MBq)	SD	Dose (mGy/MBq)	SD	Dose (mGy/MBq)	Dose (mGy/MBq)
Adrenals			6.40E−03	5.42E−04	6.42E−03	5.37E−04	8.18E−03	7.30E−03
Brain	3.20E−03	7.09E−04	1.77E−03	2.64E−04	1.77E−03	2.64E−04	2.15E−03	1.96E−03
Breasts			4.58E−03	2.99E−04	4.58E−03	2.96E−04	5.86E−03	5.22E−03
Gallbladder wall			7.01E−03	6.41E−04	7.07E−03	6.44E−04	8.69E−03	7.88E−03
LLI wall^a^	8.87E−03	2.38E−03	2.05E−02	4.03E−03	2.12E−02	4.04E−03	2.41E−02	2.27E−02
Small intestine^a^	4.52E−02	1.14E−02	3.01E−02	6.43E−03	3.03E−02	6.45E−03	3.54E−02	3.29E−02
Stomach wall^a^	6.16E−03	2.06E−03	1.13E−02	1.95E−03	1.13E−02	1.97E−03	1.36E−02	1.25E−02
ULI wall^a^	1.16E−02	3.35E−03	1.85E−02	3.78E−03	1.87E−02	3.77E−03	2.18E−02	2.03E−02
Heart wall^a^	1.41E−02	3.12E−03	1.29E−02	1.83E−03	1.29E−02	1.83E−03	1.53E−02	1.41E−02
Kidneys	2.86E−02	7.27E−03	4.63E−02	1.14E−02	4.64E−02	1.13E−02	5.06E−02	4.85E−02
Liver	5.11E−02	1.31E−02	1.55E−02	3.64E−03	1.56E−02	3.61E−03	2.08E−02	1.82E−02
Lungs	3.01E−02	3.92E−03	1.55E−02	1.90E−03	1.55E−02	1.90E−03	1.93E−02	1.74E−02
Muscle			5.28E−03	3.72E−04	5.45E−03	3.87E−04	6.88E−03	6.17E−03
Ovaries			N/A	N/A	N/A	N/A	1.04E−02	9.28E−03
Pancreas	1.43E−03	7.16E−04	1.01E−02	3.70E−03	1.01E−02	3.68E−03	1.16E−02	1.09E−02
Red marrow	1.44E−02	2.81E−03	7.12E−03	3.84E−04	7.23E−03	3.79E−04	7.98E−03	7.61E−03
Osteogenic cells			8.57E−03	4.09E−04	8.64E−03	4.10E−04	1.17E−02	1.02E−02
Skin			4.36E−03	2.95E−04	4.43E−03	2.98E−04	5.60E−03	5.02E−03
Spleen	1.08E−02	7.26E−03	2.98E−02	1.85E−02	2.98E−02	1.85E−02	3.60E−02	3.28E−02
Testes			5.34E−03	3.77E−04	5.81E−03	4.43E−04	N/A	2.91E−03
Thymus			5.24E−03	3.33E−04	5.24E−03	3.33E−04	6.68E−03	5.96E−03
Thyroid	3.99E−04	2.10E−04	9.81E−03	4.29E−03	9.81E−03	4.29E−03	1.17E−02	1.08E−02
Urinary bladder wall^a^	7.32E−02	1.51E−02	8.94E−02	1.73E−02	1.51E−01	2.99E−02	2.03E−01	1.77E−01
Uterus			N/A	N/A	N/A	N/A	1.20E−02	1.09E−02
Total body	8.77E−01	6.75E−02	6.49E−03	5.24E−04	6.68E−03	5.32E−04	8.46E−03	7.57E−03
ED ICRP60			1.57E−02	1.50E−03	1.93E−02	1.95E−03	2.43E−02	2.07E−02
ED ICRP103			1.65E−02	1.68E−03	1.98E−02	1.98E−03	2.25E−02	1.90E−02

^a^Irradiation from the organ content is accounted for residence time calculation

The standard organs receiving the highest absorbed dose were the urinary bladder wall, the kidneys, and the small intestine (89.4, 46.3 and 30.1 μGy/MBq, respectively). The measured absorbed dose in the choroid plexuses was 39.6 μGy/MBq in the male cohort and resulted to be 56.6 μGy/MBq for the female extrapolation (see Table 3).

**Table 3:** Dosimetry assessment in choroid plexuses. The mass of the choroid plexuses was estimated assuming a cylindrical geometry based on dimensions reported by Kuruoglu et al. for both male and female subjects [38]

	Residence time (hours)				Dose (mGy/MBq)	
	Average	Min	Max	SD	Male (2.76 g)	Female (1.81 g)
Choroid plexuses	3.08E−04	1.35E−04	3.66E−04	9.88E−05	3.96E−02	5.66E−02

**Fig. 4:**
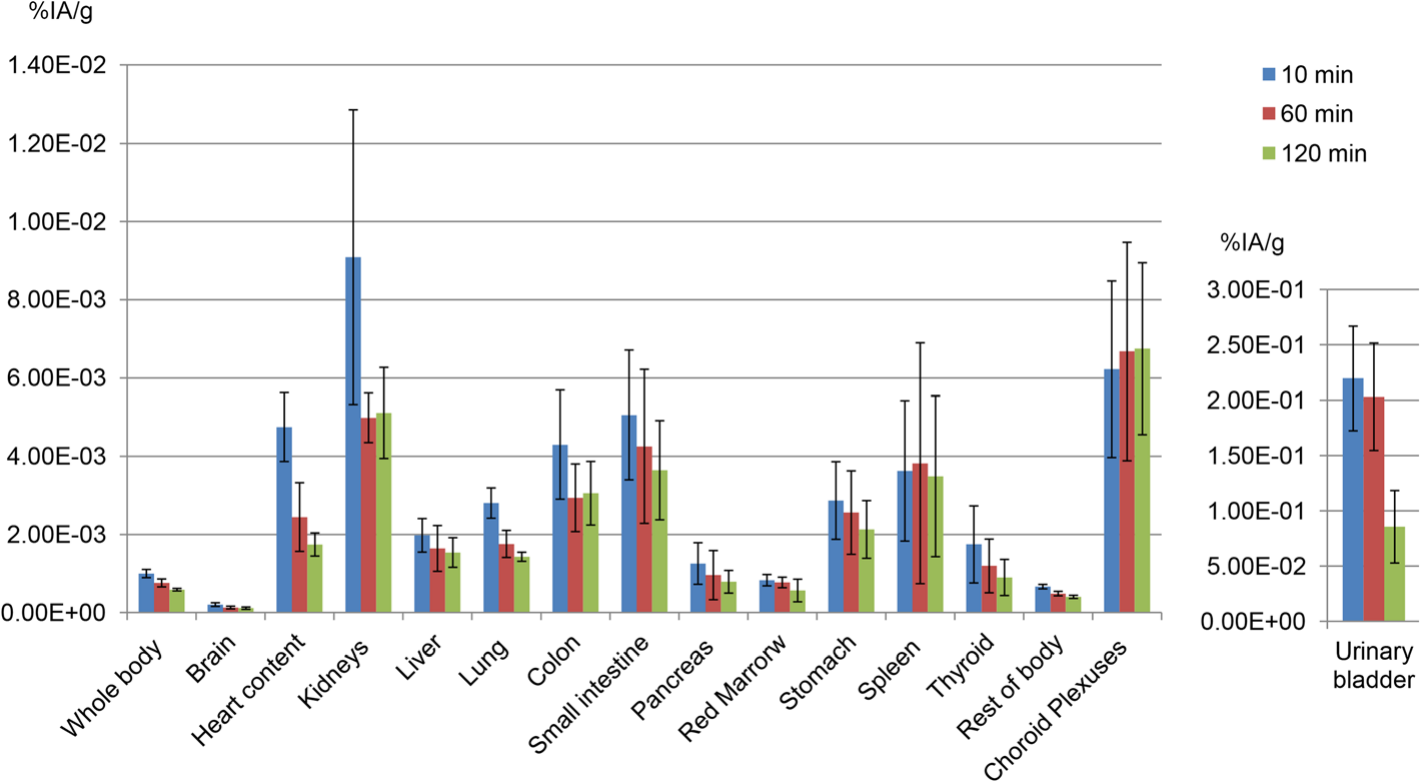
Organ time-activity curves corrected for ^68^Ga physical decay. These data essentially show the biological organ kinetics of ^68^Ga-NODAGA-RGDyK during the observation time. *Colour bars* represent the average percent of injected activity per gram of tissue (%IA/g) ± standard deviation

Organ time-activity curves corrected for ^68^Ga physical decay are shown in Fig. 4.

For a 30-min voiding cycle, the EDs were 15.7 and 16.5 μSv/MBq according to ICRP60 and ICRP103, respectively.

The extrapolation to female dosimetry resulted in organ absorbed doses 17% higher than those of male patients, on average. For female patients, the extrapolated EDs were 24.3 μSv/MBq and 22.5 μGy/MBq according to ICRP60 and ICRP103, respectively (1-h voiding cycle).

The extrapolation to 1-h voiding cycle resulted in a 74% increase of the residence time in the urinary bladder. This resulted in a 23–24% ED increment for male and female, respectively. Table 4 shows a dosimetry comparison with previous studies on RDG-based PET radiopharmaceuticals.

**Table 4:** Comparison of effective doses (ED) according to ICRP60 and absorbed doses to kidneys between available studies on RGD-based radiopharmaceuticals assuming 1-h voiding cycle. Mean ± SD are reported

Radiopharmaceutical	No. of patients	ED ICRP60 (μSv/MBq)	Dose to kidneys (μGy/MBq)
^18^F-Galacto-RGD (Beer et al.) [37]	18	18.68 ± 2.42	29.52 ± 11.95
^18^F-RGD-K5 (Doss et al.) [34]	4	15 ± 1	45 ± 10
^18^F-FPPRGD2^a^ (Mittra et al.) [33]	5	39.6 ± 18.1	97.6 ± 50.2
^68^Ga-NOTA RGD (Kim et al.) [36]	10	24.98 ± 4.39	71.61 ± 28.38
^68^Ga-DOTA-E [cRGDfK]2^a^ (Lopey-Rodriguez et al.) [35]	5	20.9 ± 5.1	53.8 ± 5.3^b^
^68^Ga-NODAGA-RGDyK (this study)	5^b^	19.3 ± 1.8^b^	46.4 ± 11.3^b^

^a^Dimeric RDG radiopharmaceuticals

^b^Values are tabulated for male patients only

## Discussion

Radiopharmaceuticals targeting integrins have a potential very broad field of application, spanning from cancer theranostics to cardiovascular diseases. Several RGD peptides have been tested over the recent past showing that the number of RDG moieties, the isotope and the chelator profoundly affect the synthetic process, binding potential and in vivo biodistribution [3]. A clear advantage of ^68^Ga-NOTA- or ^68^Ga-NODAGA RGD-based radiopharmaceuticals is that they allow for a fast and fully automated labelling at room temperature. Hence, dosimetric studies are necessary as they might inform the choice of the radiopharmaceutical, especially in view of potential therapeutic applications [40, 41].

We had previously carried out a biodistribution study of ^68^Ga-NODAGA-RGDyK in mice where projected human doses were also calculated. In addition, this previous preclinical work included a tolerance test of NODAGA-RGDyK, showing no toxicity at 1000-fold excess per kilogram of body weight compared with the maximum amount of peptide injected in a 70-kg patient [24]. In the present study, we provide the first in-human dosimetry of ^68^Ga-NODAGA-RGDyK in five male patients scheduled to undergo carotid endarterectomy. Similar-sized studies were carried out with two fluorinated compounds, the dimeric ^18^F-FPPRGD2 [33] and the monomeric ^18^F-RGD-K5 [34] respectively, as well as with the dimeric ^68^Ga-labelled DOTA-RGDfK [35]. Two larger dosimetry studies, including 18 and 10 patients each, were conducted with the monomeric ^18^F-Galacto-RGD [37] and ^68^Ga-NOTA-RGD, respectively [36].

Consistently with most of the abovementioned studies, we also found the most irradiated organs to be the urinary bladder wall and the kidneys; a prominent dose was also given to the intestine, the spleen and the liver.

Contrarily to Doss and co-authors [34], we could not confirm the gallbladder as one of the most irradiated organs, being the tracer uptake of the gallbladder not different from that of the surrounding liver parenchyma. In addition, we did not find significant dose heterogeneity between the intestinal segments, which is a clear difference with the results of Mittra et al. [33].

Dose to kidneys is of paramount importance in case of therapeutic applications, which might require the use of specific protocols for kidney protection [42]. As shown by preclinical studies, the radiotracer retention in the kidneys increases with RGD multimerization [43]. In accordance with that, we reported an absorbed dose to kidneys of 46.4 μGy/MBq, which is nearly identical to that found by Doss and colleagues with a fluorinated monomeric RGD (i.e. 45 μGy/MBq) [34] and half than that reported by Mittra et al. with a fluorinated dimeric peptide (i.e. 97.6 μGy/MBq) [33] (see Table 4). The doses to kidneys reported by others, though in the same range of values, present differences that can be explained by the different methods used. In particular, Kim et al. found higher absorbed doses (71.61 μGy/MBq) with a ^68^Ga-labelled monomeric RGD which can be considered similar to the one used in the present study; however, their methodology differs in the number of acquired time points (8 per patient) and in the fit of the activity curve, which was obtained by applying a trapezoid integration of acquired data assuming only physical decay after the last time point (75 min post injection) [36].

Beer et al. reported lower absorbed kidney doses with the most commonly used RDG-based tracer, the monomeric ^18^F-Galacto-RGD (29.52 μGy/MBq). However, they applied a partial organ segmentation on three adjacent slices, which might result in quantitative bias in presence of organ activity heterogeneity [37]. Among the available dimeric peptides, ^68^Ga-DOTA-E [cRGDfK]2 showed relatively low absorbed doses; however, a comparison with the results of this study is difficult because the number of exponential components used in the fit and the mass of the organ considered for dosimetry (patient specific or phantom) were not specified [35].

In the present study, actual measurements were based on a 30-min voiding interval and the EDs were estimated according to both the ICRP60 and the ICRP103. In addition, to allow the comparison with other similar studies, we also derived EDs for a 1-h voiding cycle (Table 4).

According to the ICRP60, the EDs for a voiding interval of 30 min were 15.7 μSv/MBq for male and 19.6 μSv/MBq for female. Interestingly, these results confirm the validity of the extrapolation we previously made from mice biodistribution data to human dosimetry [24]. In that previous study, we had estimated human EDs being 12 μSv/MBq for male and 16 μSv/MBq for female, respectively. Of note, our extrapolation of EDs from mice data showed a better agreement with human data than the extrapolation from monkeys made by Doss and co-workers (25 vs. 53% dose difference in our and in Doss’ study, respectively) [34].

By assuming a 1-h bladder voiding cycle, our ED results to be 20.7 μSv/MBq for the reference person, that is 20% higher than with 30-min voiding cycle. This dose estimation is comparable with the 19 μSv/MBq expected form a ^18^F-FDG exam [44] and would result in an ED of 3.86 mSv after the injection of 200 MBq ^68^Ga-NODAGA-RGDyK. It needs to be acknowledged that the ED estimations for the reference person are potentially biased by the absence of experimental data on female, that is an undesired effect of our dosimetry study protocol, which was approved for the first five patients only. Therefore, it was necessary to derive the female ED from the residence times obtained in the male subjects who exclusively composed our cohort.

Because of the limited number of patients, we did not calculate doses for patient-specific organ volumes. Rather, we computed doses according to OLINDA/EXM 1.0 reference phantoms. This approach is reasonable in the low dose range typical of radiopharmaceutical used for diagnostic purposes in which the patient radioprotection is the main concern, considering the fact that ED is a metric used for assessing stochastic risk in a population. On the contrary, the use of patient-specific organ masses would be crucial if a therapeutic approach was considered.

A peculiarity of this work is the estimation of the absorbed dose to the choroid plexuses, which are vascular structures localized in the brain ventricles, responsible for the production of the cerebrospinal fluid [39]. Specific uptake by the choroid plexuses was already observed with dimeric RGD peptides [33, 35], but, to the best of our knowledge, no previous studies using monomeric tracers had reported such feature. Lopez-Rodriguez and co-authors had measured the residence time in the choroid plexuses but did not provide an estimation of the absorbed dose, which was probably due to the difficulty of defining an actual volume for this organ [35]. To estimate the choroid plexuses mass, we referred to a previous magnetic resonance study [38]. We neglected the contribution of the plexuses localized in the third and fourth ventricles, as they clearly showed lower uptake than those localized in the temporal and occipital horns of the lateral ventricles (Fig. 2). Interestingly, choroid plexuses appear to be still in the biological uptake phase at the end of the acquisition time (Fig. 4). Although there are no specific dose constraints for the choroid plexuses in external beam radiotherapy, in our opinion the relatively high doses delivered to these structures would merit further consideration, especially in case of therapeutic applications of RGD peptides.

## Conclusions

We have performed the first in-human dosimetry of ^68^Ga-NODAGA-RGDyK. This study indicates that a 200 MBq injection of ^68^Ga-NODAGA-RGDyK leads to an effective dose in man of 3.86/3.92 mSv according to the ICRP60/ICRP103 recommendation. For future therapeutic applications, specific attention should be directed to delivered dose to kidneys and potentially also to the choroid plexuses.
